# Gestational Diabetes, Colorectal Cancer, Bariatric Surgery, and Weight Loss among Diabetes Mellitus Patients: A Mini Review of the Interplay of Multispecies Probiotics

**DOI:** 10.3390/nu14010192

**Published:** 2021-12-31

**Authors:** Emmanouil Benioudakis, Eleni Karlafti, Alexandra Bekiaridou, Triantafyllos Didangelos, Theodossis S. Papavramidis

**Affiliations:** 1 1st Propaedeutic Department of Internal Medicine, Medical School, University General Hospital of Thessaloniki AHEPA, Aristotle University of Thessaloniki, 54636 Thessaloniki, Greece; manolis2668@hotmail.gr (E.B.); linakarlafti@hotmail.com (E.K.); ampekiaridou@gmail.com (A.B.); didang@auth.gr (T.D.); 2 1st Propaedeutic Surgical Department, University Hospital of Thessaloniki AHEPA, Aristotle University of Thessaloniki (AUTH), 54636 Thessaloniki, Greece

**Keywords:** probiotics, diabetes mellitus, surgical patients, wound healing, weight loss, bariatric surgery

## Abstract

Diabetes mellitus has been steadily increasing over the past decades and is one of the most significant global public health concerns. Diabetes mellitus patients have an increased risk of both surgical and post-surgical complications. The post-surgical risks are associated with the primary condition that led to surgery and the hyperglycaemia per se. Gut microbiota seems to contribute to glucose homeostasis and insulin resistance. It affects the metabolism through body weight and energy homeostasis, integrating the peripheral and central food intake regulatory signals. Homeostasis of gut microbiota seems to be enhanced by probiotics pre and postoperatively. The term probiotics is used to describe some species of live microorganisms that, when administered in adequate amounts, confer health benefits on the host. The role of probiotics in intestinal or microbial skin balance after abdominal or soft tissue elective surgeries on DM patients seems beneficial, as it promotes anti-inflammatory cytokine production while increasing the wound-healing process. This review article aims to present the interrelation of probiotic supplements with DM patients undergoing elective surgeries.

## 1. Introduction

Diabetes mellitus (DM) has been rising rapidly in recent decades and is one of the largest global public health concerns [1,2]. The global prevalence of DM is estimated to be 9.3%, projecting an increasing trend for the coming decades [1]. DM mainly consists of three types: diabetes mellitus type 2 (T2D), which accounts for approximately 90% of the total DM incidents; diabetes mellitus type 1 (T1D), which requires lifelong administration of exogenous insulin; and gestational diabetes mellitus (GDM) [1]. Uncontrolled DM creates an increased risk of serious macro- and micro-vascular complications, resulting in kidney failure, blindness, and increased mortality. It is also frequent among surgical patients, constituting a major burden for postoperative recovery.

Abdominal surgeries, major or minimal, are a wide category of surgeries that include all gastrointestinal, urological, and gynaecological operations undertaken for any indication [3]. Despite the scientific advancement of laparoscopic abdominal surgeries, they are still associated with a high rate of severe postoperative complications and long-term disability [4,5]. DM patients have an increased risk of both surgical and post-surgical complications [5]. The post-surgical risks are associated both with the primary condition that led to surgery and the hyperglycaemia itself. Hyperglycaemia may occur in both DM and non-DM patients due to the stress of the surgery operation and is closely associated with postoperative infections, such as wound infection, sepsis, and pneumonia [6]. The administration of probiotics has demonstrated interesting results, improving postoperative outcomes among DM patients. The term probiotics is used to describe some species of live microorganisms that, when administered in adequate amounts, confer health benefits on the host [7] due to their contribution to the intestinal microbial balance [8]. Naturally, the human gastrointestinal tract harbours a vast group of microorganisms known as “gut microbiota” [6]. The gut microbiota is a complex micro-ecosystem of billions of bacteria, fungi, viruses, and bacteriophages [6]. It seems to contribute to glucose homeostasis through bacteria involved in the control of inflammation and energy homeostasis [9] as well as through the host’s metabolism [10]. 

The increasing global concern towards postoperative infections and the limitations of infection control strategies has led to alternative strategies. Nutritional adjuncts, such as probiotics, have emerged as potential complementary treatments [11]. The interactions between probiotics and metabolic diseases as well as the underlying mechanisms remain unclear [12]. This review aims to highlight the effects of multispecies probiotic supplements and their interplay on gestational diabetes, colorectal cancer, bariatric surgery, and weight loss among DM patients.

## 2. Search Strategy 

Literature research was performed on the association of multispecies probiotics with DM. The literature research was carried out in September of 2021 on Elsevier and Medline databases: PubMed, ScienceDirect, and Scopus. Searches were carried out using the following keywords: (1) multispecies probiotics; (2) colorectal cancer, colon cancer; (3) bariatric surgery; (4) weight loss; (5) diabetes mellitus, gestational diabetes, type 1 diabetes, type 2 diabetes; and (6) surgical site infection, wound healing. These keywords were entered into the above databases using Boolean logic. The studies included had to have a double-blind randomized design, include DM, and be written in English. Papers were excluded if only the abstract was retrieved, they were preprints, or were conference proceedings.

## 3. Results and Discussion

### 3.1. Probiotics on Gestational Diabetes Mellitus

Gestational diabetes mellitus (GDM) is a type of diabetes that is diagnosed in the second or third trimester of pregnancy [13] and remains a common and increasing complication of pregnancy [14]. Women with GDM are at risk of high blood pressure, labour induction, or unnecessary caesarean delivery [15,16]. In addition, GDM is associated with infant risks, such as macrosomia, respiratory distress syndrome, birth injuries, bone fractures and shoulder dystocia, jaundice, and hypoglycaemia, thus endangering the child’s health [17]. Obesity is closely associated with GDM, contributing to nearly half of GDM’s prevalence [18]. Obesity has become a global healthcare problem, with steadily increasing rates among women of the reproductive age [19]. Simultaneously, it remains one of the few modifiable risk factors for GDM [14]. Bariatric surgery is becoming increasingly common for the achievement of weight loss [20], while the studies suggest that reductions in body weight before pregnancy may hold the key to the prevention of GDM [21,22]. This section will present the interrelation of probiotics with weight loss and insulin sensitivity among GDM women. 

Initially, Halkjae et al. [23], in a double-blind placebo-controlled study conducted among 50 obese pregnant women, showed that the administration of probiotics strains with *Streptococcus thermophilus*, *Bifidobacterium,* and *Lactobacillus* genera did not significantly contribute to the reduction and control of body weight. Nevertheless, probiotics appear to influence metabolism, body weight, and energy homeostasis as they integrate the peripheral and central food intake regulatory signals [24]. Furthermore, Wickens et al., in a double-blind, randomized, placebo-controlled parallel trial conducted among 423 obese pregnant women, showed that mean blood glucose levels were significantly lower among the participants administered *Lacticaseibacillus rhamnosus* as well as *Lacticaseibacillus rhamnosus* being associated with lower rates of GDM in women aged ≥35 years [25]. The authors speculated that *Lacticaseibacillus rhamnosus* altered the gut microbiota composition and function, improving insulin sensitivity and inflammation in the host. [25]. Tay et al. investigated the effect *Lacticaseibacillus rhamnosus* had among 33 participants with pre-DM [26]. The probiotic supplement of *Lacticaseibacillus rhamnosus*, administered for 12 weeks, showed a reduction of most DM-related values, such as haemoglobin A1c and weight, compared to the baseline but not between the intervention and control group. Nevertheless, the probiotic supplement seems to improve social functioning and mental health outcomes, a finding that needs to be further explored. Finally, according to Davidson et al.’s systematic review, the administration of probiotics for preventing GDM appears to increase the risk of pre-eclampsia and may increase the risk of hypertensive disorders of pregnancy, albeit the data were heterogeneous [27]. Further research on the clear-cut benefits and risks of probiotics, especially on pregnant women, is warranted. Clinical trials of probiotics on gestational diabetes mellitus are presented in Table 1.

### 3.2. Probiotics Administration on DM Patients Undergoing Bariatric Surgery 

Bariatric surgery constitutes an effective treatment for morbid obesity and causes significant weight loss results [28]. It has the potential to reduce the risks of developing GDM when performed before pregnancy [29]. The most common and effective bariatric surgery procedures for reducing weight are the Roux-en-Y gastric bypass and the vertical sleeve gastrectomy. The data suggest that probiotic administration to bariatric surgery patients provides glycaemic improvements and better DM control [30]. 

Mokhtari et al., in their placebo-controlled, double-blind, randomized clinical trial, investigated the use of modulators of gut microbiota, such as probiotic supplements, on blood markers of endotoxin and lipid peroxidation in patients undergoing Roux-en-Y gastric bypass surgery [28]. Participants with T2D (*n* = 6) were randomly assigned to the probiotic or control croup (1:1). The initiation of the treatment was pre-operative. Each probiotic capsule contained seven species of probiotic bacteria (*Streptococcus thermophilus*, *Lacticaseibacillus casei*, *Lacticaseibacillus rhamnosus*, *Lactobacillus acidophilus*, *Lactobacillus delbrueckii* subsp. *bulgaricus*, *Bifidobacterium breve,* and *Bifidobacterium longum*), while placebo capsules contained maltodextrin. The results showed that a four-month consumption of probiotic supplements prohibited an elevation in levels of the lipopolysaccharide-binding protein (LBP) and contributed to a more significant weight loss compared to placebo consumption. When the impact on LBP was adjusted for weight loss, this effect disappeared. The measurement of LBP is considered to reflect the circulating lipopolysaccharides (LPS) status. The literature suggests that obesity is associated with increased LPS and LPB levels [31], while high levels of LPS are observed among T1D and T2D patients [32,33,34]. The alteration in the gut microbiome has a pivotal role in the initiation and maintenance of obesity-related inflammation. Gut microbial metabolites can trigger innate immunity, especially LPS, through a complex process that activates the pro-inflammatory cytokines [20,34]. Furthermore, probiotic bacteria have also been shown to promote anti-inflammatory cytokine production, accelerating the inflammation phase, balancing intestinal microbial composition, and regulating immune-related cytokine expression [35,36]. Thus, the influences of probiotics can explain the effects on weight loss due to alterations in the composition of gut microbiota.

Subsequently, Ramos et al., in a randomized placebo-controlled, double-blind clinical trial, investigated the use of *Lactobacillus acidophilus* and *Bifidobacterium lactis* in the early postoperative period of patients undergoing Roux-en-Y gastric bypass operation [37]. Among the participants (*n* = 110), 13.8% of them had a diagnosis of T2D. The observed occurrence of T2D between probiotic or control group was (1.2:1). The present study showed significant anthropometric and metabolic parameters improvements in both groups after the Roux-en-Y gastric bypass operation. In addition, a significant reduction in triglyceride levels and total cholesterol were found in the probiotic group. Probiotic supplementation causes a decrease in cholesterol through multiple and not well-known mechanisms [37,38]. 

Subclinical chronic inflammation is a known complication of obesity that supports the development of T2D [39,40,41], while inflammation that occurs after bariatric surgery mitigates some of these comorbidities [42]. The levels of inflammatory biomarkers, such as Tumour Necrosis Factor α (TNF-α), Interleukin-6 (IL6), and C-reactive protein (CRP), can change rapidly due in part to the tight regulation of cytokine production [41,42,43]. There are no data to support the concept that probiotics might be a valuable tool to counteract chronic inflammation in healthy older adults [44]. However, probiotic supplementations seemed to significantly affect inflammatory parameters [30,45,46]. Clinical trials of DM patients undergoing bariatric surgery are presented in Table 2.

### 3.3. Colorectal Cancer, DM Comorbidity, and Probiotics

The chronic inflammation caused by the breach of the balance barrier between beneficial and harmful bacteria in the gastrointestinal (GI) tract disrupts the intestinal homeostasis, increasing the risk of colorectal cancer (CRC) and T2D development [47]. CRC is one of the most diagnosed cancers globally [48], with an increasing number of cases in recent decades [49]. In addition, T2D was associated with an increased incidence and prevalence of CRC, as hyperglycaemia is thought to have a significant role in the proliferation and expansion of colorectal cancer cells [50]. The influence of probiotic supplements on CRC is twofold. Multiple studies have suggested that probiotics, especially *Lactobacillus* strains, can be used to prevent CRC. Gut microbiota directly or indirectly influences the epigenetic processes in the aetiology of CRC, as it is associated with beneficial factor effects on glycaemic control [51,52,53]. The benefit of probiotic supplements in glycaemic control was described in a meta-analysis conducted by Yao et al., in which 12 randomized control trials were evaluated with a total population of 684 T2D patients [54]. The meta-analysis demonstrated that probiotic supplementation significantly reduced glucose levels and alleviated insulin resistance. Surgery remains one of the main treatments of primary or metastatic colorectal cancers, and probiotics do not constitute a possible treatment [55,56]. 

Gut microbiota has an essential role in postoperative infectious complications [57], such as surgical site infection (SSI), which is very common after colorectal procedures [58]. The benefit of gut microbiota after colorectal cancer surgery was reported by Kotzampassi et al. [57] in a double-blind, placebo-controlled randomized study. It included 164 patients who had undergone elective, open, colonic resection with primary anastomosis. Of the participants, 25% had comorbid T2D diagnosis and were placed in either the placebo or the probiotics perioperative treatment group (1:1.05). The probiotic supplements consisted of four probiotic strains: *Lactobacillus acidophilus*, *Lactiplantibacillus plantarum*, *Bifidobacterium lactis,* and *Saccharomyces boulardii*. The probiotic supplements, or the placebo, respectively, were first administered on the day of operation and for 14 consecutive days. The results showed a considerable reduction in the overall major postoperative complications and infectious complications as well as reduced hospitalization in the probiotic group. Finally, a positive correlation between the gene expression of SOCS3 with the inflammatory biomarkers was reported in the probiotic group. The mechanism of action of probiotics may be related either to earlier bowel movement, thus preventing bacterial translocation from the gut, or to modulation of the innate immune responses [57].

Zaharuddin et al. [59] described the benefits of probiotic supplements in a randomized, double-blind placebo-controlled trial. The use of a mixture of six viable probiotic supplements strains, including *Lactobacillus acidophilus*, *Lactobacillus delbrueckii* subsp. *lactis*, *Lacticaseibacillus casei*, *Bifidobacterium longum*, *Bifidobacterium bifidum*, and *Bifidobacterium infantis,* among post-surgical patients with colorectal cancer was investigated. Fifty-two patients were included in the study, of whom 7.6% were diagnosed with comorbidity of T2D and were placed between the probiotic supplements and the placebo group (1:1). Participants were instructed to consume the product four weeks after their surgeries, twice daily, for six months. The results showed reduced levels of pro-inflammatory cytokines in the probiotic supplements group. This result was mainly related to lactic acid strains, as evidence has revealed that this probiotic can interfere with the signalling pathways, thus affecting the level of cytokines production.

Finally, Golkhalkhali et al. [60] investigated the effect of the probiotic supplements *Lactobacillus acidophilus*, *Lacticaseibacillus casei*, *Lactobacillus delbrueckii* subsp. *lactis*, *Bifidobacterium bifidum*, *Bifidobacterium longum,* and *Bifidobacterium infantis* in tandem with omega-3 fatty acid supplementation on the quality of life, chemotherapy side effects, and inflammatory markers among CRC patients. The study was conducted on 140 participants who were instructed to consume the probiotic supplements or placebo for four weeks. Among the participants, 13.5% had a comorbid diagnosis of DM (the type is not specified) and were placed between the probiotic supplements and the placebo group (1.7:1). The results showed an improvement in the probiotic supplements group in the quality-of-life parameters, such as physical function, emotional functioning, nausea, vomiting, and fatigue. Furthermore, eight weeks from baseline, the inflammatory cytokine IL-6 was significantly reduced in the treatment group compared to the placebo group. Clinical trials of colorectal cancer, diabetes mellitus, and probiotics are presented in Table 3.

### 3.4. Probiotic Supplements and DM Rat Models

Animal models of human diseases allow us to examine the role of environmental and genetic factors during disease development and to develop new therapeutic strategies, albeit lacking in human disease complexity [61]. Recent research has shown that alterations in the gut microbiota because of probiotic supplements directly affect the inflammatory state in humans. Many animal-model studies that focused on DM have clearly demonstrated the beneficial effects of probiotic strains [62]. Patients with DM usually have impairment in healing due to an imbalance of the wound-healing process [63]. The wound-healing process includes haemostasis, inflammation, and proliferative and remodelling procedures [64]. DM affects one or more biological mechanisms of these processes, caused by many factors, such as hyperglycaemia, chronic inflammation, and micro- and macro-circulatory dysfunction [64,65]. Diabetic foot ulcers are among the most common and severe complications of DM, leading to alarming figures of amputation and disability [66]. This section will present the beneficial effect of probiotic supplements raised from animal models with DM on wound healing after surgery.

Campos et al. investigated the effects of probiotic supplements on skin wounds in diabetic rats [63]. The DM rats (*n* = 46) started the preoperative probiotic or placebo supplementation eight days before surgery. Probiotic supplements were administered orally within the evidence-based recommendations for humans. The supplements consisted of *Lacticaseibacillus paracasei*, *Bifidobacterium lactis*, *Lacticaseibacillus rhamnosus,* and *Lactobacillus acidophilus*. Wounds were macroscopically evaluated a few days after the surgery. The results showed improved wound healing, mature collagen deposition, neovascularization stimulation, a reduction of the inflammatory process, and improvement of glycaemic control in the probiotic supplement group. The anti-inflammatory activity in a dextran sulfate sodium (DSS)-induced colitis rat model was also shown in Dai et al.’s [67] research, with the use of *Streptococcus thermophilus*, *Bifidobacterium longum*, *Bifidobacterium breve*, *Bifidobacterium infantis*, *Lactobacillus acidophilus*, *Lactiplantibacillus plantarum*, *Lacticaseibacillus casei,* and *Lactobacillus delbrueckii* subsp. *bulgaricus*. In addition, Mohtashami et al. [68] evaluated the wound-healing potentials of probiotics on diabetic cutaneous wounds after surgery in rats (*n* = 27). *Lactobacillus delbrueckii* subsp. *bulgaricus* and *Lactiplantibacillus plantarum* probiotics were administered topically [68]. Probiotics have demonstrated some benefits at the skin level through oral and topical administration [68]. The results showed that during 14 days of treatment, probiotics improved the diabetic wounds as well as showing alterations in levels of inflammatory cytokines.

Rat model experiments have proved that probiotics can be beneficial in a wide range of DM physiochemical parameters. Obesity is a high-risk factor for GDM and T2D [23,28,69]. Weight reduction with probiotic supplements in rats has been studied by Okediya et al. [70]. This study, among obese rats (*n* = 15), showed that probiotic supplements *Lactobacillus acidophilus* and *Lactiplantibacillus plantarum*, when administered orally, were effective in reducing the weight of obese rats. In addition, *Lactiplantibacillus plantarum* was proven effective in weight loss in Yang et al.’s [71] research. In a similar vein of DM physiochemical parameters improvement was Hsieh et al.’s [12] research among 50 DM rats, orally administered *Liquorilactobacillus salivarius*, *Lactobacillus johnsonii*, *Limosilactobacillus reuteri*, and *Bifidobacterium animalis* subsp. *lactis* once a day for eight weeks. The results showed a significant improvement in glucose tolerance, glycaemic levels, insulin levels, and a reduction in insulin resistance, while in streptozotocin (STZ)-induced DM rats, an attenuated STZ-induced β-cell death in a dose-dependent manner was observed, with pancreatic regions remaining intact. In addition, there was a decrease in blood urea and lipid levels, including low-density lipoprotein, triglycerides, and total cholesterol. Trials of DM rats are presented in Table 4.

### 3.5. Limitations of the Current Review

There are several limitations in our review that should be considered. Initially, the characterization of «probiotics» did not occur through the standardized methods but after the reported studies. There are a limited number of DM related studies as well as a different methodology to them. Finally, studies presented in the Chapter: 3.3. «Colorectal cancer, DM comorbidity and probiotics» included patients with comorbid DM, so the results presented in this chapter followed the main outcomes of those studies, unless otherwise reported.

## 4. Conclusions

Overall, probiotics are generally considered safe for consumption. Exceptions are women who are already pregnant, as theoretically, there are potential risks, such as pre-eclampsia, increased risk of hypertensive disorders of pregnancy, and a negative influence on hematologic values in infants, such as haemoglobin levels [27,72]. Results are limited and in many cases contradictory. Future research is needed to confirm the overall safety of probiotics during pregnancy. The use of probiotics appears to contribute to weight management, thus indirectly preventing the development of DM and GDM, which are closely correlated with increased body weight. Probiotics also induce improvement in physicochemical parameters and metabolic control, so they can be considered a therapeutic approach for T2D. Their effectiveness, along with diet management, can make a significant contribution to weight loss. The gut microbiota, the skin bacteria colonization, and the microbial balance through probiotic supplement administration have been proven essential for postoperative abdominal and soft tissue surgical procedures. DM has an increased risk of serious micro- and macro-vascular complications, leading to wound-healing impairment. DM patients are at greater risk of being trapped in a persistent inflammatory condition with elevated levels of pro-inflammatory cytokines. Probiotics have an essential effect on rehabilitation after surgical procedures among DM patients, as they demonstrate anti-inflammatory effects.

## Figures and Tables

**Table 1:** Clinical trials of probiotics on gestational diabetes mellitus.

Study/Ref	Method/ Timeline/ Sample/	Probiotics Used	Results
Halkjae et al. (2020) [24]	RDBPCT2 capsules twice daily for 14–20 Weeks *n* = 50	Streptococcus thermophilus Bifidobacterium breve Bifidobacterium longum Bifidobacterium infantis Lactobacillus acidophilus Lactobacillus plantarum Lactobacillus paracasei Lactobacillus delbrueckii subsp. bulgaricus	slight increase in α-diversity in the probiotic group Probiotic supplements contributed to the reduction and body weight control
Wickens et al. (2017) [26]	RDBPCT 1 capsule daily for up to 12 months *n* = 423	Lactobacillus rhamnosus	Lactobacillus rhamnosus was associated with lower rates of GDM in women aged ≥35 years mean blood glucose levels were significantly lower in the probiotic group
Tay et al. (2020) [27]	RDBT 12-week *n* = 33	Lactobacillus rhamnosus	Probiotic supplements: significantly reduced HbA1c and weight from baseline significantly improved social functioning and mental health

Abbreviations: RDBPCT, randomized double-blind placebo control trial; RDBT, randomized double-blind trial; GDM, Gestational diabetes mellitus.

**Table 2:** Probiotics administration to diabetes mellitus patients undergoing bariatric surgery (clinical trials).

Study/ Ref	Method/ Timeline/ Sample/	Probiotics Used	Results
Mokhtari et al. (2019) [29]	RDBPCT 1 capsule per day for 4 months and 9 months of additional follow-up *n* = 46	Streptococcus thermophilus Lactobacillus casei Lactobacillus rhamnosus Lactobacillus acidophilus Lactobacillus bulgaricus Bifidobacterium breve Bifidobacterium longum	Probiotics supplements significantly improve: serum LBP TNF-α vitamin B12 vitamin D3 weight loss
Ramos et al. (2021) [38]	RDBPCT 2 capsule per day for 3 months *n* = 110	Lactobacillus acidophilus Bifidobacterium lactis	Probiotics supplements: significantly decrease cholesterol significantly increase vitamin D significantly increase Vitamin B12 levels

Abbreviations: RDBPCT, randomized double-blind placebo control trial; LBP, lipopolysaccharide-binding protein; TNF-α, Tumour Necrosis Factor alpha.

**Table 3:** Colorectal cancer, diabetes mellitus comorbidity, and probiotics (clinical trials).

Study/Ref	Method/ Timeline/Sample	Probiotics Used	Results
Kotzampassi et al. (2015) [58]	RDBPCT 1 capsule twice a day for 15 days *n* = 164	Lactobacillus acidophilus Lactobacillus plantarum Bifidobacterium lactis Saccharomyces boulardii	Probiotic supplements: significantly decreased the rate of all postoperative major complication significantly reduced the rate of postoperative pneumonia significantly decreased surgical site infections significantly decreased anastomotic leakage
Zaharuddin et al. (2019) [60]	RDBPCT 1 capsule twice a day for 6 months *n* = 52	Lactobacillus acidophilus Lactobacillus lactis Lactobacillus casei Bifidobacterium longum Bifidobacterium bifidum Bifidobacterium infantis	Significantly reduced levels of pro-inflammatory cytokines: TNF-α IL-6 IL-10 IL-12 IL-17A IL-17C IL-22 in the probiotic supplements group.
Golkhalkhali et al. (2017) [61]	RDBPCT 1 capsule per day for 4 weeks *n* = 140	Lactobacillus acidophilus Lactobacillus casei Lactobacillus lactis Bifidobacterium bifidum Bifidobacterium longum Bifidobacterium infantis	Improvement in the probiotic supplements group: Quality-of-Life parameters (physical function, emotional functioning, nausea, vomiting and fatigue) IL-6 was significantly reduced

Abbreviations: RDBPCT, randomized double-blind placebo control trial; RDBT, randomized double-blind trial; TNF-α, Tumour Necrosis Factor alpha; IL-6, Interleukin 6; IL-10, Interleukin-10; IL-12, Interleukin-12; IL-17A, Interleukin-17A; IL-17C, Interleukin-17C; IL-22, Interleukin-22.

**Table 4:** Probiotic supplements and in-vivo models of DM rats.

Study/Ref	Method/ Timeline/ Procedure Type/Sample	Probiotics Used	Results
Hsieh et al. (2021) [13]	Probiotics vs control Probiotics administered orally once per day for 8 weeks Observational study *n* = 50 DM rats	Lactobacillus salivarius Lactobacillus johnsonii Lactobacillus reuteri Bifidobacterium animalis subsp	The probiotic supplement group significantly improved: Glucose tolerance Glycaemic levels Insulin levels Insulin resistance (HOMA-IR)
Campos et al. (2020) [64]	Probiotics vs control Probiotics administered orally eight days before surgery and for 18 days total Skin wound *n* = 64 DM rats	Lactobacillus paracasei Bifidobacterium lactis Lactobacillus rhamnosus Lactobacillus acidophilus	The probiotic supplement group showed: Improved wound healing mature collagen deposition neovascularization stimulation reduction of the inflammatory process improvement of glycemic control
Mohtashami et al. (2020) [70]	Probiotics vs control/ Topical administration of probiotics for 14 days Cutaneous wounds *n* = 27 DM rats	Lactobacillus bulgaricus Lactobacillus plantarum	Treatment with probiotics: Accelerated the healing process of diabetic wounds Modulated the inflammatory cells in wound sites

Abbreviations: DM, diabetes mellitus; HOMA-IR, Homeostatic Model Assessment for Insulin Resistance.
